# Mg_3_Al_2_Si_3_O_12_ jeffbenite inclusion in super-deep diamonds is thermodynamically stable at very shallow Earth’s depths

**DOI:** 10.1038/s41598-022-27290-9

**Published:** 2023-01-03

**Authors:** Fabrizio Nestola, Mauro Prencipe, Donato Belmonte

**Affiliations:** 1 Dipartimento di Geoscienze, Università degli Studi di Padova, Via Gradenigo 6, 35131 Padua, Italy; 2 Dipartimento di Scienze della Terra, Università degli Studi di Torino, Via Valperga Caluso 35, 10125 Turin, Italy; 3 Dipartimento di Scienze della Terra, dell’Ambiente e della Vita, Università degli Studi di Genova, Corso Europa 26, 16132 Genoa, Italy

**Keywords:** Geophysics, Mineralogy

## Abstract

Jeffbenite (having the same chemical composition of pyrope, ~ Mg_3_Al_2_Si_3_O_12_, and also known as TAPP phase) is a mineral inclusion only found in diamonds formed between about 300 and 1000 km depth) and is considered a stable phase in the transition zone (410–660 km depth) and/or in the shallowest regions of the lower mantle (around 660–700 km depth). This rare and enigmatic mineral is considered to be a pressure marker for super-deep diamonds and therefore it has a key role in super-deep diamond research. However, the pressure–temperature stability fields for Mg_3_Al_2_Si_3_O_12_ jeffbenite is unknown and its actual formation conditions remain unexplored. Here we have determined the thermodynamic pressure–temperature stability field for the jeffbenite Mg-end member and surprisingly discovered that it is stable at low pressure–temperature conditions, i.e., 2–4 GPa at 800 and 500 °C. Thus, Mg_3_Al_2_Si_3_O_12_ jeffbenite is not the high-pressure polymorph of pyrope and is likely a retrogressed phase formed during the late ascent stages of super-deep diamonds to the surface.

## Introduction

Jeffbenite (ideal formula Mg_3_Al_2_Si_3_O_12_) is a rare mineral that so far was only found as mineral inclusion in super-deep diamonds^1^. It was discovered in 1997^2,3^ and since then it has been referred to as TAPP, an acronym from “**T**etragonal **A**lmandine **P**yrope **P**hase” for its stoichiometry, which is coincident with that of the pyrope-almandine garnet series. However, the crystal structure of jeffbenite is different from that of garnet, thus garnet and jeffbenite are actually polymorphs. In 2016, TAPP was finally given a mineral name approved by IMA, which is “jeffbenite” (IMA 2014–097)^1^ to honour Jeffrey W. Harris and Ben Harte, two eminent experts in the field of diamond research. From its first discovery 26 years ago, only 23 natural jeffbenites were reported in literature and 7 of them were identified only by chemical analysis; 2 further jeffbenites reported in the literature are synthetic. So, only 16 natural jeffbenite inclusions in super-deep diamonds have been identified by X-ray diffraction and/or micro-Raman spectroscopy (see Table 1, which summarizes all jeffbenites reported so far in the literature). Despite its rarity, jeffbenite inclusions in diamonds are considered to be an indicator of a super-deep origin for the diamond hosts and therefore it is an important mineral. Its super-deep origin is indeed well accepted in literature and jeffbenite is generally considered a transition zone or lower mantle mineral by the diamond research community^1–20^.

**Table 1:** Chemical composition (in wt% oxides), method of identification and name of inclusions of all jeffbenites reported so far in literature.

References	This study	1		2			3			4				
Identification method	Calculated	Diffraction, Raman	Chemical composition	Diffraction			Diffraction			Diffraction				
Name of inclusion	Ideal Mg_3_Al_2_Si_3_O_12_	IMA approved	In the same diamond	BZ206B	BZ207A	BZ244B	BZ243A	BZ259A1	BZ259A2	BZ238A	BZ243A			
SiO_2_	44.72	41.74	36.05	42.43	39.56	42.12	42.24	42.24	41.83	41.41	42.24			
TiO_2_	–	0.06	3.56	0.01	4.20	0.06	0.04	0.03	0.02	0.03	0.04			
Al_2_O_3_	25.28	23.84	17.82	23.48	20.16	23.83	24.17	23.12	23.15	23.33	24.17			
Cr_2_O_3_	–	2.86	0.01	2.22	1.39	2.80	2.41	2.38	2.40	2.99	2.41			
FeO	–	4.59	20.12	4.64	9.41	4.60	5.19	4.45	4.43	1.29	1.76			
Fe_2_O_3_	–	–	–	–	–	–	–	–	–	4.10	3.81			
MnO	–	0.79	0.37	0.47	0.25	0.96	0.90	0.67	0.65	0.92	0.90			
MgO	30.00	25.16	17.99	26.66	24.85	25.63	24.36	26.01	26.91	24.95	24.36			
CaO	–	0.09	0.04	0.12	0.03	0.09	0.11	0.10	0.11	0.13	0.11			
Na_2_O	–	0.10	0.10	0.15	0.03	0.09	0.09	0.15	0.15	0.16	0.09			
K_2_O	–	–	0.04	–	–	–	–	–	0.01	0.02	–			
NiO	–	–	–	0.07	0.03	0.01	0.02	0.02	0.01	0.01	0.02			
total	100.00	99.23	96.10	100.25	99.91	100.19	99.53	99.17	99.67	99.34	99.91			

References	5	6	7	10	11	13				15		17	19	
Identification method	Chemical composition	Diffraction	Diffraction	Chemical composition	Diffraction	Chemical composition				Raman		Diffraction	Raman	
Name of inclusion	KK-83a	BZ240B	BZ205A	J4	Synthetic	13	66	C14	C40	SL-13	SL-80	Synthetic	Ju5-102	Ju5-117
SiO_2_	38.90	42.65	42.54	35.17	38.57	39.10	46.89	36.26	35.82	39.90	41.90	34.39	35.75	37.21
TiO_2_	2.22	0.01	0.02	4.09	3.61	5.33	0.24	4.29	4.53	5.37	1.41	–	4.03	3.29
Al_2_O_3_	18.99	23.91	23.88	19.92	19.03	18.34	9.63	13.66	16.51	18.80	21.80	0.31	16.68	16.84
Cr_2_O_3_	3.68	2.34	2.47	0.03	3.15	1.33	0.02	–	0.03	1.31	0.25	–	0.04	0.04
FeO	6.87	4.76	4.96	23.10	–	8.85	22.48	14.61	24.51	9.00	10.10	15.48	22.85	19.93
Fe_2_O_3_	–	–	–	–	8.89	–	–	–	–	–	–	32.07	–	–
MnO	0.06	0.74	0.84	0.49	–	0.18	0.48	0.58	0.53	0.16	0.12		0.42	0.49
MgO	26.86	25.84	25.43	15.91	25.33	24.84	18.92	29.60	18.40	25.40	24.40	18.63	17.38	19.43
CaO	0.54	0.11	0.11	0.05	–	0.01	0.11	–	0.08	0.07	0.05	–	0.08	0.10
Na_2_O	0.02	0.12	0.16	0.05	–	0.06	0.39	0.51	0.22	0.05	0.02	–	0.11	0.13
K_2_O	–	–	–	–	–	–	0.01	–	0.01	0.01	0.03	–	–	0.01
NiO	0.02	0.01	0.01	–	–	–	–	–	–	0.03	0.07	–	–	–
total	98.16	100.49	100.42	98.81	98.58	98.04	99.17	99.51	100.63	100.10	100.15	100.88	97.34	97.47

Ref.^20^ reported a Raman-identified Ti–rich jeffbenite; however no chemical analysis was reported in that work.

However, excluding a very Ti–rich synthetic jeffbenite^11^, at present no pressure–temperature stability fields of jeffbenite are published and this mineral remains a geological enigma: (1) at what depth in the mantle does jeffbenite actually form? (2) is the Mg-end member of jeffbenite a higher- or lower-pressure polymorph of pyrope garnet?

To answer these questions, here we constrain the pressure–temperature stability field of pure jeffbenite, Mg_3_Al_2_Si_3_O_12_. As no thermodynamic data for jeffbenite are available in literature, we compute the data from first principles, at the hybrid Hartree–Fock/Density Functional Theory (HF/DFT) level, within the limit of the quasi-harmonic approximation and in the framework of statistical thermodynamics. This allows us to comprehend the actual nature of the pure Mg end-member of jeffbenite.

## Results

### Jeffbenite versus pyrope molar volume: an evident discrepancy

Indeed, the present work was driven not only by the need to constrain the pressure–temperature stability field for a mineral taken to be characteristic of super-deep diamonds, but also because already in 1997^2^ study of the first natural jeffbenite (at that time indicated as TAPP) revealed inconsistencies in terms of volume and density with respect to garnet. In detail, the natural jeffbenite discovered in 1997^2^ (sample 244B, on which the authors refined the crystal structure) had an approximate composition equal to [Mg_2.64_Fe^tot^_0.27_(Ca + Na + Mn)_0.08_][Al_1.85_ Cr_0.15_][Si_2.91_Al_0.09_]O_12_ and a Mg# [Mg/(Mg + Fe)] = 0.91; its unit-cell volume was *V* = 774.35(± 0.77) Å^3^. Such unit-cell volume can be converted in a molar volume equal to 11.657(± 0.012) J/bar. Comparing this molar volume with that of a garnet with similar composition along the pyrope-almandine series [Mg_2.70_Fe_0.30_]Al_2_Si_3_O_12_ and Mg# = 0.91 (e.g., see Table 3 of ref.^21^), it is evident that the molar volume of the garnet, which is equal to 11.332(± 0.001) J/bar, is significantly smaller than that of jeffbenite.

This first simple calculation in terms of molar volume shows a strong discrepancy: the molar volume of jeffbenite seems significantly larger than its pyrope polymorph, thus jeffbenite should not be the higher-pressure polymorph of pyrope; this said on the basis of simple thermodynamical considerations.

To confirm this unexpected result with respect to what is believed in the super-diamond research, we need a complete set of thermodynamic parameters.

### Thermoelastic properties, entropy and Gibbs free energy of jeffbenite

We have determined a Birch-Murnaghan equation of state truncated to the third order (BM3-EOS^22^) for jeffbenite, which provides the following values of unit-cell volume, *V*_0_, bulk modulus, *K*_0T_ and first pressure derivative, *K*´(at *T* = 298.15 K):

$$V_{0} = 766.033\;{\text{\AA}}^{3}$$

$$K_{0T} = \, 175.39\;{\text{GPa}}$$

$$K^{\prime } = 4.09$$

(*V*_0_ can be expressed in J/bar providing a value of 11.532, which is already rescaled by a 0.9787 factor to take into account the typical overestimation from the DFT calculation. The correction factor was estimated starting from the same identical overestimation on pyrope). The reason for the overestimation of the cell volume in ab initio calculations (at the DFT or HF/DFT level of the theory) is well understood^23,24^ and it has long been proved to be *not* an issue in the estimation of the second derivatives of the energy versus volume function on which, in turn, bulk moduli and vibrational frequencies are computed.

The full elastic constant tensor of jeffbenite have been computed at the static level (i.e. *T* = 0 K, *P* = 0 GPa and no zero point effects included) by fitting the second derivatives of the energy with respect to strain components, then using stress–strain relations^25^. Jeffbenite (tetragonal, space group *I*$\overline{4}$2*d*) has six independent elastic stiffnesses, calculated as follows: C_11_ = C_22_ = 319.2 GPa; C_12_ = 140.7 GPa; C_13_ = C_23_ = 123.5 GPa; C_33_ = 257.0 GPa; C_44_ = C_55_ = 100.5 GPa; C_66_ = 129.1 GPa. The aggregate elastic moduli (bulk and shear moduli) inferred by the elastic tensor through a Voigt-Reuss-Hill averaging scheme are *K*_VRH_ = 184.2 GPa and G_VRH_ = 98.4 GPa, respectively. The former value is in excellent agreement with that obtained at *T* = 0 K and *P* = 0 GPa from the static BM3-EOS (i.e. *K*_0_ = 182.8 GPa), which supports the internal consistency of ab initio elastic data computed for jeffbenite in this work.

**Figure 1:**
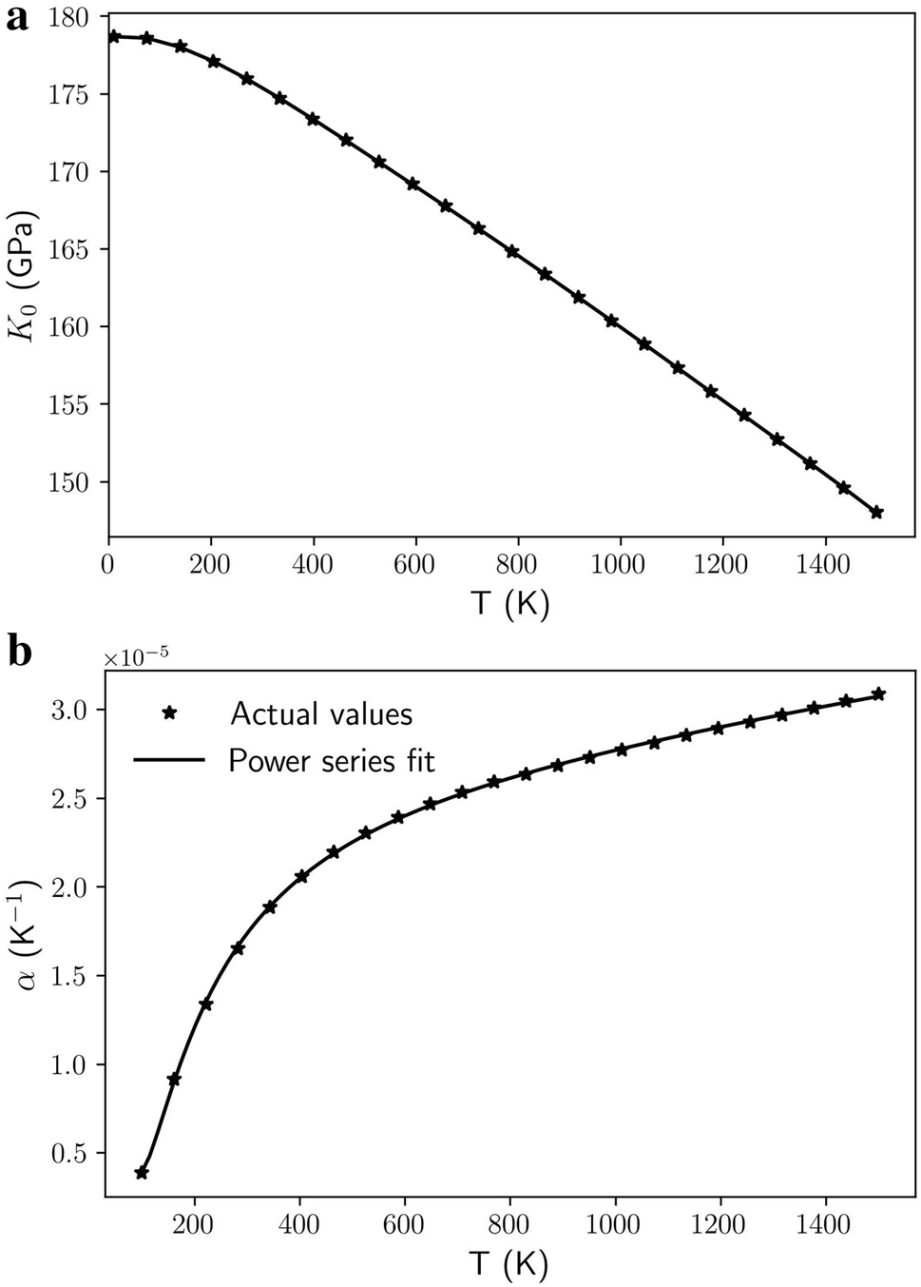
Dependency of bulk modulus, *K*_0_ (in GPa) (**a**) and that of the volume thermal expansion coefficient, α (in K^−1^) (**b**) as a function of temperature, *T* (in K), for jeffbenite in this study.

The evolution of the bulk modulus as a function of temperature is shown in Fig. 1a. The temperature dependency of the bulk modulus is expressed as:

$$dK_{0T} /dT = \, - 0.0200\;{\text{GPa}}/{\text{K}}$$

The volume thermal expansion coefficient is given by:

$$a_{0V} = \, 1.717 \, \times \, 10^{ - 5} {\text{K}}^{ - 1} \left( {{\text{at}}\;298.15\;{\text{K}}} \right)$$

The thermal expansion coefficient evolution as a function of temperature is shown in Fig. 1b.

The values of entropy and Gibbs energy of formation (starting from pyrope) are given as:

$$S_{0} = \, 253.36\;{\text{J}}/{\text{mol}}\;{\text{K}}$$

$$DG_{0} = \, - 13360.85\;{\text{J}}/{\text{mol}}$$

### Calculated versus experimental Raman spectrum of jeffbenite

**Figure 2:**
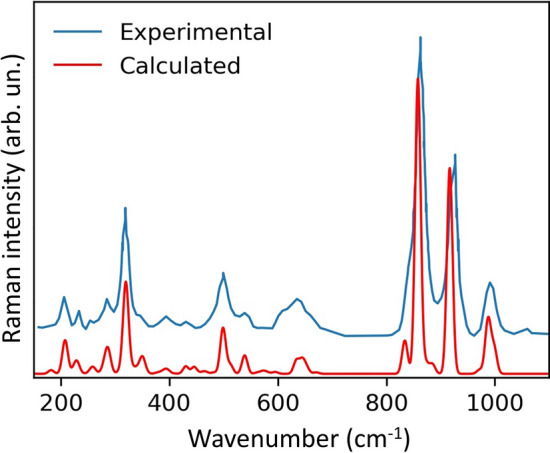
Comparison between the calculated Raman spectrum of jeffbenite form this study and the experimental one^1^.

To show that the thermodynamic properties of jeffbenite calculated in this work are reliable, we have compared the Raman spectrum calculated from our data with an experimental one for the holotype jeffbenite approved by IMA (Fig. 2)^1^. The spectra are nearly identical, with the calculated one showing a higher resolution (this is quite typical as it is unlikely that an experimental spectrum can reach the resolution of the computed one); the experimental spectrum appears to be slightly shifted toward higher wavenumbers: this could be related to the differences in chemical composition between the pure Mg_3_Al_2_Si_3_O_12_ jeffbenite used for the calculation in this work and the natural sample, which shows an average of 4.6 wt% of FeO (of which about 20% as Fe^3+^), some Cr^3+^ and slightly less Al than the pure end member. However, although such limited chemical differences, the overlap is satisfactory.

As the entropy (at least the vibrational contribution to it, which is the only one if order/disorder phenomena are excluded, as it is in the present case) is uniquely determined by the phonon spectrum, the excellent match between the calculated and the experimental Raman spectrum in Fig. 2 provides assurance that the value of entropy we determined for jeffbenite is reliable (see next section).

### Gibbs free energy, entropy and pressure–temperature stability field of jeffbenite

To analyse the possible phase transition between the two polymorphs jeffbenite and pyrope we need first to compute the Gibbs energy. The differences in Gibbs energy between jeffbenite and pyrope, as functions of pressure, at different (*fixed*) temperatures is described as follows:

$$\Delta G\left( P \right) = G_{jeff} \left( P \right) - G_{py} \left( P \right)$$

The free energy of pyrope has been evaluated in several different ways:

1. Full quantum–mechanical evaluation;
2. Quantum–mechanical evaluation but with a correction for the entropy at standard conditions ($S_{0}$) taken from the H&P 2011 database
3. From the H&P 2011 database
4. From the H&P 2002 database
5. From the Stixrude database

**Figure 3:**
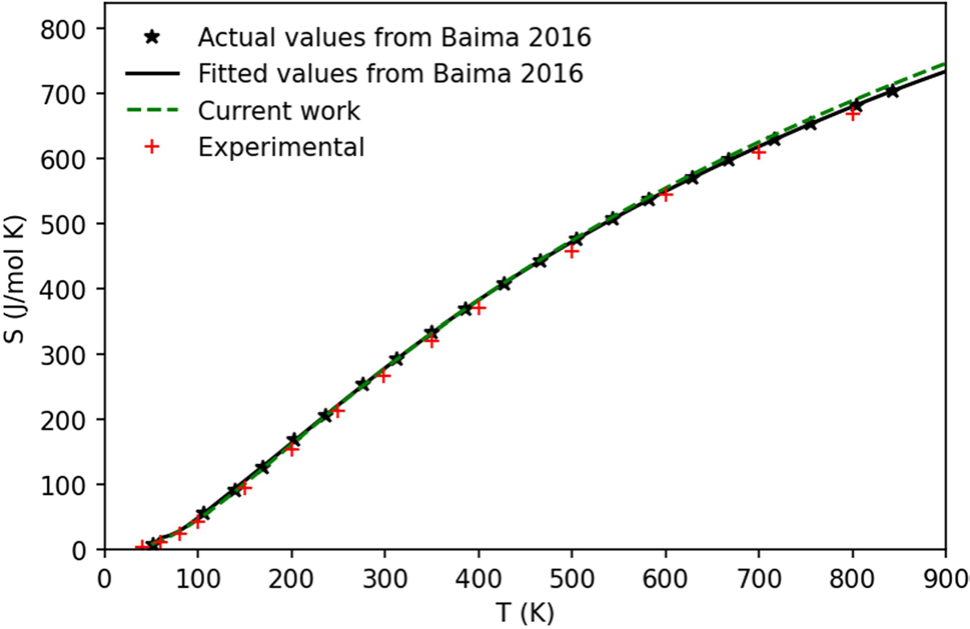
Entropy, S (in J/mol K), as a function of temperature, *T* (in K), for the pure end member pyrope (the experimental values are from ref.^30^ up to 350 K and from ref.^31^ for data at higher temperatures).

The entropy of pyrope at standard conditions is a critical parameter affecting the pressure of transition from jeffbenite to pyrope as the temperature increases. The quantum–mechanical evaluation of *S*_0_ is 276.31 J/mol K [the modified-Kieffer model^26^], has been used for the evaluation of the acoustic mode contribution, with frequencies taken from the original Kieffer publication^27^; the value of the entropy without such acoustic contribution is significantly lower: 263.82 J/mol K, about 4.5% lower]. The value of *S*_0_ we determined in this work by quantum–mechanical evaluation is nearly identical to a previous one^28^ (276.85 J/mol K), at the ab initio* level*, through a super-cell approach for the evaluation of both the phonon dispersion effects and the acoustic contribution. Furthermore, the vibrational entropy values calculated in this work for pyrope are consistent with previous computational investigations performed at the hybrid HF/DFT level^29^. In Fig. 3, the entropy as functions of temperature, at zero pressure and from different works, is shown.

The difference between the entropies from Baima et al.^28^ and the current work are negligible, at least up to a temperature of 600 K. Experimental values are generally lower than the corresponding ab initio values. Indeed, the experimental $S_{0}$ is 266.27 J/mol K. The values of $S_{0}$ for pyrope adopted in thermodynamic databases are: 266.30, 269.50 and 242.36 J/mol K for HP02^32^, HP11^33^ and Stx^34^, respectively.

**Figure 4:**
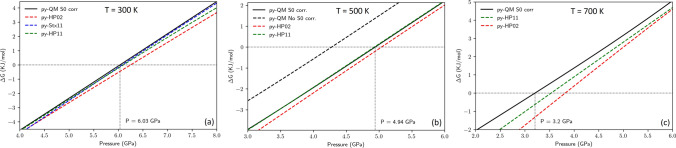
Δ*G* (in KJ/mol) for jeffbenite in this study as a function of pressure, *P* (in GPa), at *T* = 300 K (**a**), at *T* = 500 K (**b**) and 700 K (**c**).

In this work, the Gibbs energy of jeffbenite was evaluated by using the same methods and computational parameters as those employed for pyrope. In Fig. 4, a comparison of the $\Delta G\left( P \right)$ between the two polymorphs, at 300 (Fig. 4a), 500 (Fig. 4b) and 700 K (Fig. 4c) is shown. In Fig. 4, the straight line $\Delta G = 0$ (*zero line*) marks the transition from jeffbenite to pyrope, that occurs at 6.02 GPa at 300 K (Fig. 4a), as seen from the intersection of the solid line with the *zero line*. In this case, the Gibbs energy of pyrope is evaluated at the ab initio level. Dashed lines, in colour, refer to the evaluation of the Gibbs energy of pyrope by means of the thermodynamics databases HP02, HP11 and Stx. The estimated transition pressures are 6.25 GPa (HP02), 6.11 GPa (HP11) and 6.08 (Stx). At this relatively low temperature (300 K), the impact of entropy on the computed $\Delta G$ is almost negligible. At higher temperatures, the situation significantly changes: in particular, at a temperature of 500 K (Fig. 4b), the transition pressure decreases to 4.27 GPa (red dashed line).

However, by recognising the fact that the entropy at standard conditions (*S*_0_) of pyrope, computed at the ab initio level, is overestimated with respect to the experimental value, a correction could be applied that results in black solid line of the Fig. 4b (*S*_0_ corr., which refers to such a correction of the entropy in the standard state). The correction here adopted corresponds to the HP11 value of *S*_0_. In this case, the transition pressure is 4.94 GPa. Indeed, the higher value of the transition pressure in the latter case is due to a relative decrease of the Gibbs energy of pyrope (in turns, due to the decrease of its entropy). The transition pressures computed by employing the Gibbs energies of pyrope from the databases are 5.04 (HP02) and 4.93 GPa (HP11). The curve resulting from the Stixrude database is not reported, as the corresponding value of *S*_0_ for pyrope is too far from the experimental one to be considered reliable; in addition, at variance with the entropy reported in the other databases, with the experimental measurements and with the quantum–mechanical estimations, the Stixrude database reports a value for *S*_0_ that is lower than the corresponding value for jeffbenite computed in the present work; this leads to an increase of the transition pressure as the temperature is increased (6.45 GPa, at 500 K; this *P*–*T* point is not represented in Fig. 4b).

At higher temperatures (see Fig. 4c for T = 700 K) the observed trends are confirmed. The estimation of the transition pressure, if pyrope is dealt at the ab initio level (with the correction for *S*_0_ described above), is 3.20 GPa; otherwise, the pressure is 3.53 GPa (pyrope from the HP11 database) or 3.82 GPa (pyrope from the HP02 database). Even with the Stixrude database, at 700 K, the transition pressure decreases (5.92 GPa; again, this P–T point this is not shown in Fig. 4c).

**Figure 5:**
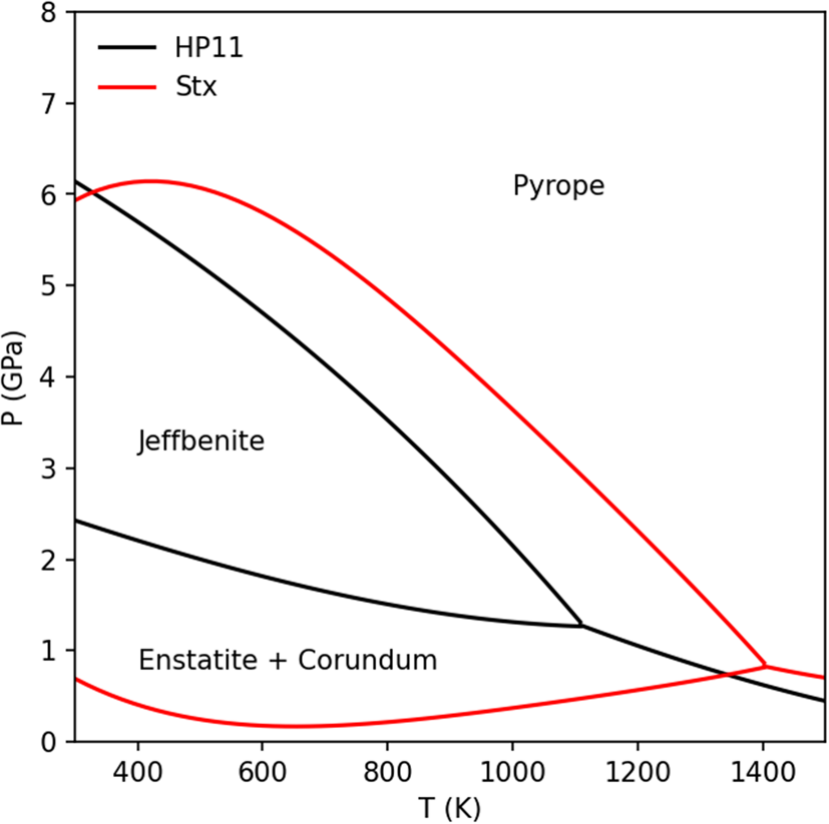
Pressure–temperature stability field of the pure Mg end-members jeffbenite and pyrope calculated using Perple_X^35^.

By entering the thermodynamic data of Mg_3_Al_2_Si_3_O_12_ jeffbenite determined in this work into the Stixrude database^34^ as implemented in Perple_X software^35^, the phase diagram section for the Mg_3_Al_2_Si_3_O_12_ system can be calculated and is shown in Fig. 5. Surprisingly, and in total disagreement with respect to the literature, the stability field indicates that jeffbenite is a low pressure polymorph of pyrope; in detail, by using the Stixrude database^34^, jeffbenite stability field expands toward higher temperatures and lower pressures. At the maximum temperature, e.g., 1400 K, jeffbenite is stable at about 0.90 GPa, while it reaches a maximum of 6 GPa just above 400 K.

## Discussion

### Thermoelastic properties: a comparison between jeffbenite and pyrope

The main target of this work was to compute pressure–temperature stability field of jeffbenite and to establish its polymorphic relationship with pyrope. However, thermodynamic and thermoelastic data of jeffbenite were lacking in literature and thus we had to calculate them.

With respect to pyrope, in term of bulk modulus, jeffbenite shows a value of 175.39 GPa which lies within the average value through several data published in literature for pyrope, that spans between about 164 and 182 GPa, with an average value around 170.2 GPa^25,36–47^. The first pressure derivative, *K*´, is 4.09 for jeffbenite and appears to be slightly lower than the average value of all published values for pyrope, which is 4.63 (ranging between 3.2 and 6.4). The computed bulk modulus and *K´* for pyrope (same computational parameters as those employed for jeffbenite) are 162.8 GPa and 4.36, respectively, and are consistent with the ranges of the experimentally measured values.

In terms of bulk modulus dependency with temperature, we obtained for jeffbenite a value equal to -0.020 GPa/K against an average (experimental) value of − 0.022 GPa/K of pyrope [although this value has a quite significant data scatter in literature going from − 0.0194(30)^43^, to − 0.021(9)^48^, up to − 0.026(4)^47^]. However, the computed *dK*_0_*/dT* for pyrope is -0.033 GPa/K; that is, it is slightly higher from that of jeffbenite.

The volume thermal expansion coefficient for jeffbenite is here calculated as *α*_0V_ = 1.717 × 10^–5^ K^-1^ (at 298.15 K) and, differently with respect to what we observed for the bulk modulus, we find a significant difference between jeffbenite and pyrope, with this last showing an average value of 2.19 × 10^–5^ K^-1^ (with values ranging between about 2 and 2.5 × 10^–5^ K^-1^, ref.^44,49,50^). The ab initio computed value of *α*_0V_ for pyrope is 3.0 × 10^–5^ K^-1^ (at 298.15 K).

### Pressure–temperature stability field of jeffbenite

Thanks to the above calculated thermodynamic data, here we have reported, for the first time, the pressure–temperature stability field of jeffbenite, at least for its Mg end-member with composition Mg_3_Al_2_Al_3_O_12_, which represents the ideal formula of jeffbenite reported in the official mineral list updated to July 2022 by the International Mineralogical Association. The stability field definitively indicates that jeffbenite is not a high pressure mineral and is the lower pressure polymorph of pyrope (see Fig. 5).

Although, based on what is now well accepted in literature, jeffbenite is considered only as a super-deep inclusion in diamonds and thus the higher pressure polymorph of pyrope, our results contradict this statement and, at the same time, they are totally consistent with the analysis of the molar volumes of the two phases. Indeed, as we also mentioned in the first section of the Results, even without any of our calculation and completely neglecting our work, a clear contradiction was already evident at the experimental level by comparing the molar volume of jeffbenite, which has *V*_0_ = 11.532 (J/bar), and that of pyrope, which has a *V*_0_ = 11.316 (J/bar)^51^.

**Figure 6:**
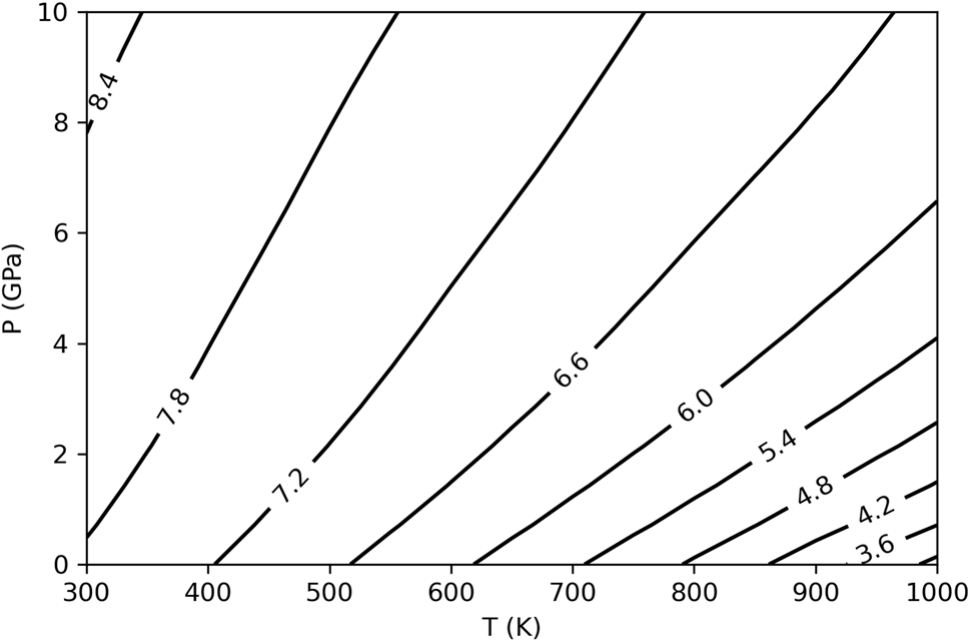
Primitive unit cell volume difference (in Å^3^) between jeffbenite and pyrope, in the indicated pressure–temperature ranges.

By taking into account the thermoelastic parameters of jeffbenite and pyrope as calculated in this work, jeffbenite has a larger molar volume with respect to pyrope in the whole P–T stability range investigated in this work (see Fig. 6). This definitively demonstrates that the former cannot be a higher pressure polymorph of the Mg_3_Al_2_Si_3_O_12_ end-member phase but instead it is the lower pressure polymorph. This is consistent with our pressure–temperature stability field for jeffbenite in Fig. 5.

As to the other thermodynamic properties, it is interesting to note that some attempts was made in the past to empirically estimate the thermodynamic properties of jeffbenite in such a way to reconcile the phase relations observed in type III inclusions in diamonds from Brazil with an hypothetical stability field of this garnet phase^52,53^. In particular, a minimum pressure and temperature of 25 GPa and 2273 K were suggested for the formation of jeffbenite on the basis of observations on the Ca content of type III inclusions in diamond where jeffbenite coexists with two silicate perovskites^52^. Nevertheless, the purely hypothetical HP-HT stability field of jeffbenite in the predicted phase diagram for the enstatite (MgSiO_3_)—pyrope (Mg_3_Al_2_Si_3_O_12_) join, besides not being supported by any experimental evidence, is clearly flawed by physical unsoundness of the thermodynamic data assumed for jeffbenite. In fact, the former thermodynamic assessment^53^ was forced to assume a much higher compressibility of jeffbenite with respect to pyrope as well as huge entropy values for jeffbenite to stabilize this phase at high pressures and temperatures, respectively. This clearly contradicts our first principles results, which define both internally- and physically-consistent thermoelastic parameters and reliable entropy values for jeffbenite and pyrope as well. As an example, the entropy value assessed for jeffbenite at *T* = 970 K^53^ is *S*_0 970_ = 827.35 J/mol·K, which is overestimated by roughly 10% as compared to our ab initio value (i.e. *S*_0 970_ = 754.5 J/mol·K). This discrepancy is well beyond the level of confidence by which DFT is able to predict vibrational entropy of silicate minerals^54^.

Jeffbenite is a rare silicate only found in super-deep diamonds. Although jeffbenite is a rare mineral, however, it covers a crucial role in super-deep diamond research as it is considered a very high pressure mineral marker^2^ stable at least at the transition zone depths between 410 and 660 km. However, before our study, no pressure–temperature stability fields for jeffbenite was published (with the exception of one very Ti–rich synthetic jeffbenite^11^) because no thermodynamic data were available for such rare mineral. Here we have calculated all thermoelastic and thermodynamic data and determined the first pressure–temperature stability field for the Mg jeffbenite end-member. Of the 16 natural analysed jeffbenite known in literature, well 8 jeffbenites show a Mg# between 0.90 and 0.92, 7 jeffbenites have a Mg# between 0.81 and 0.89 and only one jeffbenite shows a Mg# equal to 0.43. Thus, such data indicate that the Mg jeffbenite end-member is the most abundant and critical one to understand the behaviour of jeffbenite and this is why we focused on it.

Very surprisingly, our results definitively show that the Mg end-member of jeffbenite is not a high-pressure polymorph of pyrope and is stable at low pressure and temperature conditions (Fig. 5). However, although several Mg-rich jeffbenites were indicated by authors as super-deep inclusions, using our data on pure Mg_3_Al_2_Si_3_O_12_ jeffbenite, we cannot speculate about the thermodynamic stability of Fe-richer and Ti-richer jeffbenites (and in some jeffbenites even the Cr content could be significant); actually, for Fe-rich and Ti–rich jeffbenites authors reported synthesis experiments demonstrating that they can be obtained at high pressure (Ti–rich up to 13 GPa^11^, Fe-dominant jeffbenite at 15 GPa^17^).

Combining our results and those from experiment laboratories^11,17^, we would suggest to be cautious in using extremely Mg-rich and extremely Ti-poor jeffbenites to claim super-deep origin for their diamond hosts.

## Methods

### Computational details

Structures (unit cell parameters and atomic fractional coordinates), static energies and vibrational frequencies at the Γ point of the Brillouin zone of jeffbenite were computed at different values of the primitive unit cell volume, in the [359, 406 Å^3^] range (10 points in the range). The full elastic tensor of jeffbenite, at the equilibrium static volume was also computed. The calculations were performed at the ab initio level by using the CRYSTAL17 code^55^. The hybrid Hartree–Fock/Density Functional WC1LYP^56^^,^^57^^,^^58^^,^^59^ was employed. The localized basis sets chosen for the atoms were of the type 85-11G(1d) for Mg, 85-11G(2d) for Al, 88-31G(2d) for Si, and 8-411G(2d) for O. The thresholds controlling the computation of the Coulombic and exchange integrals (ITOL1 to ITOL5 in the CRYSTAL17 input^56^) were set to 9, 9, 9, 9 and 22. The shrinking factor (IS) controlling the sampling of points in the BZ where the electronic Hamiltonian is diagonalized was set to 4, resulting in 13 independent **k** points in the BZ. An XXL grid^56^ for the numerical evaluation of the integrals of the DFT functionals of the electron density was chosen, which corresponded to 219,069 points in the unit cell; the very high accuracy of such numerical evaluation can be measured by the integration of the electron density over the unit cell, resulting in 399.999958 electrons in the cell, out of 400. Quantum–mechanical results are included in the manuscript related files.

By using the QM-thermodynamic software^59^, which implements a standard statistical thermodynamics formalism, in the limit of the Quasi-Harmonic Approximation (QHA), vibrational frequencies and static energies at each unit cell volume were employed to compute (*i*) the Equation of State (third-order Birch-Murnaghan) parameters (*V*_0_, *K*_0_, and *K*´) at 298.15 K; (*ii*) the dependence of *K*_0_ by the temperature (d*K*_0_/d*T*); (*iii*) the specific heat at constant pressure (*C*_P_) and its temperature dependence; (*iv*) the entropy at standard conditions (*S*_0_); (*v*) the Gibbs free energy at standard conditions (*G*_0_); (*vi*) the thermal expansion and its temperature dependence. A correction to *C*_P_, *S*_0_ and *G*_0_, in order to take into account the contribution of the acoustic phonons to those quantities, was made by employing the modified Kieffer-model as described in previous work^26^. This method allows to define shear and longitudinal seismic velocities along different propagation (and polarization) directions of the single crystal from the ab initio elastic constant tensor by solving the Christoffel determinant. A set of directionally-averaged seismic velocities are then used to calculate the acoustic contributions to thermodynamic properties according to a sine wave dispersion relation assumed for the three acoustic branches in the phonon spectrum^27^.

Thermodynamics quantities were estimated up to a temperature of 1700 K, and a maximum pressure of about 20 GPa at a temperature of 298.15 K (24 GPa at 1700 K). Results are provided with the deposited files (see the below **Data availability** statement).

In order to consistently compare the stability of jeffbenite with that of pyrope, in the appropriate P/T region, identical ab initio calculations at the same HF/DFT level, which employed the same parameters as those already set for jeffbenite, where also performed for the pyrope case. The Gibbs free energy of jeffbenite at standard conditions (*G*_0_), in the appropriate scale, was computed from the difference of the ab initio energies of jeffbenite and pyrope (energies estimated at the same ab initio level) and rescaled to the *G*_0_’s found in the thermodynamic databases used for subsequent computations^33,60^. The Perple_X program^35^ was then used to compute pseudosections for fixed global stoichiometries of the system corresponding to several significant mineral assemblages.

## Data Availability

All data generated during this study are included in this published article. In detail, we deposited the following 9 files: (1) Frequencies.pdf = vibrational frequencies of jeffbenite as a function of the unit cell volume and the static pressure as a function of the primitive unit cell volume. (2) jeff_pvt_eosfit.dat = contains the volume-pressure–temperature data. (3) jeffbenite.eos = includes all thermoelastic parameters and has a format readable by EoSFIT-inc free software (http://www.rossangel.com/text_eosfit.htm). (4) R1_jeffbenite_WC1LYP_elastcon_P = 0.mb = zero-pressure static elastic constants tensor calculated for jeffbenite at WC1LYP level of theory (*C*_*ijkl*_, in Mbars). (5) R2_jeffbenite_WC1LYP_VRH_P = 0.txt = the aggregate elastic moduli (bulk modulus *K*, shear modulus *G*, Young’s modulus *E*, in Mbars; Poisson’s ratio) and longitudinal and shear seismic velocities (*V*_P_ and *V*_S_, in km/s) of jeffbenite, computed according to the Voigt-Reuss-Hill scheme^61^. (6) R3_jeffbenite_WC1LYP_elastcon_P = 0_VpG_S1S2.txt = the longitudinal and shear seismic velocities (*V*_P_, *V*_S1_ and *V*_S2_, in km/s) along any direction of propagation (and polarization) in the single-crystal of jeffbenite, obtained by solving the Christoffel determinant^26,62^. Longitudinal and shear seismic velocities calculation of jeffbenite were calculated using CAREWARE package^63^. (7) Input_file_calc.pdf = input file including the complete processing of the ab-initio data to produce the thermodynamic parameters, by means of the Qm-Thermodynamic Python program (https://qm-thermodynamics.readthedocs.io/en/main/). ^59^ .This file allows any readers to reproduce all results obtained in this work. (8) jeffbenite_WC1LYP_elastcon_P = 0.d12 = input file of zero-pressure elastic constant tensor calculation of jeffbenite at WC1LYP level of theory. (9) jeffbenite_WC1LYP_elastcon_P = 0.txt = output file of zero-pressure elastic constant tensor calculation of jeffbenite at WC1LYP level of theory calculated with CRYSTAL.
