# Author Correction: Mg_3_Al_2_Si_3_O_12_ jeffbenite inclusion in super-deep diamonds is thermodynamically stable at very shallow Earth’s depths

**DOI:** 10.1038/s41598-023-30111-2

**Published:** 2023-02-17

**Authors:** Fabrizio Nestola, Mauro Prencipe, Donato Belmonte

**Affiliations:** 1 Dipartimento di Geoscienze, Università degli Studi di Padova, Via Gradenigo 6, 35131 Padua, Italy; 2 Dipartimento di Scienze della Terra, Università degli Studi di Torino, Via Valperga Caluso 35, 10125 Turin, Italy; 3 Dipartimento di Scienze della Terra, dell’Ambiente e della Vita, Università degli Studi di Genova, Corso Europa 26, 16132 Genoa, Italy

Correction to: *Scientific Reports* https://doi.org/10.1038/s41598-022-27290-9, published online 03 January 2023

The Acknowledgments section in the original version of this Article was incomplete.

“F.N. thanks the ERC Starting Grant n. 307322.”

now reads:

“F.N. thanks the ERC Starting Grant n. 307322. D.B. warmly acknowledges financial support by the Italian MIUR PRIN2017 (Project Number 2017KY5ZX8).”

The original Article has been corrected.

